# Influence of Normo- and Hypogonadal Condition, Hyperuricemia, and High-Fructose Diet on Renal Changes in Male Rats

**DOI:** 10.1155/2017/1623597

**Published:** 2017-02-15

**Authors:** Jimena Soutelo, Yanina Alejandra Samaniego, Elsa Zotta, María Cecilia Fornari, Carlos Reyes Toso, Osvaldo Juan Ponzo

**Affiliations:** ^1^Department of Physiology, Medicine School, University of Buenos Aires (UBA), Buenos Aires, Argentina; ^2^Endocrinology Service Medical Complex, Argentine Federal Police (PFA), Churruca-Visca Hospital, Buenos Aires, Argentina; ^3^Laboratory Fornari-Bioalpha, Buenos Aires, Argentina

## Abstract

*Background*. There is a gender disparity in the incidence, prevalence, and progression of renal disease. The object of this paper is to evaluate the presence and type of renal lesion in normogonadic and hypogonadic male rats in a mild hyperuricemia induced condition and exposed to a high-fructose diet*. Methods*. 56 adult male Wistar rats were used. Animals were divided into two groups, one normogonadic (NGN) and one hypogonadic (HGN), and each group was divided into four subgroups in accordance with the treatment: control with only water (C), fructose (F), oxonic acid (OA), and fructose + oxonic acid (FOA). Renal changes were evaluated by measuring glomerulosclerosis, fibrosis, and arteriolar media/lumen (M/L) ratio. *Results*. The OA and FOA groups presented significantly hypertension (*p* < 0.001). The OA group significantly increased (*p* < 0.05) the percentage of glomerulosclerosis as well as the FOA group (*p* < 0.001). When comparing NGN versus HGN, we observed a trend to a lower glomerulosclerosis in the latter. A higher arteriolar M/L ratio was observed in the OA (*p* < 0.05) and FOA (*p* < 0.001)*. Conclusion*. Hyperuricemia conditions and a high-fructose diet favor blood pressure increase together with changes in the arteriolar media/lumen ratio and renal glomerular damage. These changes were more apparent in normogonadic animals.

## 1. Introduction

There is a gender disparity regarding the incidence, prevalence, and progression of renal disease. Most of the experiments with male animals have shown acceleration in the progression of renal lesion, compared to female animals. Moreover, in studies where manipulation of sexual hormones was applied, it was observed that female sexual hormones slow down the progression of renal disease, whereas male hormones speed it up [1]. Some epidemiological studies show controversial data [2–5], but some others prove a greater progression in men compared with women at reproductive age but not at menopausal stage, which shows a protective action by estrogens [3]. Likewise, there is a gender disparity in uric acid plasmatic levels and prevalence of gout [6]. Moreover, a significant increase in uric acid levels has been reported in postmenopausal women that receive testosterone and also in those androgenized women due to gender change [7]. Also, a decrease in uric acid level has been observed in men with chemical castration due to prostate cancer who are not treated with testosterone therapy [8]. On the other hand, evidence shows that a high-fructose diet may increase the onset of cardiovascular disease, metabolic syndrome, diabetes type 2, and renal disease [9–12]. Hyperuricemia can also foster the presence of the said conditions [13–17].

The object of this paper is to determine the presence and type of renal damage, in mild hyperuricemia and high-fructose diet, depending on testosterone levels, in normogonadic and hypogonadic male rats, and whether both conditions potentiate kidney injury.

## 2. Materials and Methods

### 2.1. Animals

Fifty-six male adult Wistar rats from the Department of Physiology, School of Medicine, University of Buenos Aires, were used for this experiment. Animals were housed in a light, temperature, and humidity controlled environment (lights on from 07.00 am to 07.00 pm, *T* 22–24°C) and were fed *ad libitum*, having access to chow and water during the experiment. The experiment began when the animals were 70 days old. Animal experiments and handling were performed according to the “Ethical principles and guidelines for experimental animals” of the Swiss Academy of Medical Sciences (3rd Edition 2005).

#### 2.1.1. Experimental Design

Eight groups of adult male Wistar rats (*n* = 7/group) were studied over a period of 5 weeks: four normogonadics (NGN) and four hypogonadics (HGN).

The NGN groups were divided into four subgroups: (a) control group (C): fed with a standard commercial diet and water; (b) fructose group (F): fed with the same diet plus 10% (w/v) fructose (Tate&Lyle, USA) in the drinking water for 5 weeks; (c) oxonic acid group (OA) (Sigma Aldrich 156124, St. Louis, MO, USA): fed with a standard commercial diet and water and receiving the uricase inhibitor OA by intragastric gavage (750 mg/kg BW, daily) [18]; and (d) fructose and oxonic acid group (FOA): fed with the control diet plus 10% (w/v) fructose in the drinking water for 5 weeks and receiving also the oxonic acid by intragastric gavage (750 mg/kg BW, daily), during the same period.

In the second group (HNG), adult male rats were orchidectomized bilaterally through an anterior median incision in the scrotum and each ductus deferens was isolated, ligated, and cut, allowing the testicle to be removed. One month after this, the HNG animals were conducted in the experimental period and were divided into the same four subgroups that received the same treatment as the four NGN groups: (a) control group (C), (b) fructose group (F), (c) oxonic acid group (OA), and (d) fructose and oxonic acid group (FOA).

In all control and fructose without OA groups, animals received water vehicle administered by intragastric gavage. In such a way, all animals had the same level of stress by gavage.

### 2.2. Body Weight and Systolic Blood Pressure Measurements

Body weight was measured daily. Amount of beverage consumed in each group of rats was calculated and adjusted daily according to the volume of liquid consumed.

Systolic blood pressure (SBP) was measured in conscious rats by a validated volume-based tail-cuff method connected to an amplifier and a data acquisition system (Rat Tail System; Innovators in Instrumentation, Landing, NJ, USA). All animals were preconditioned for blood pressure measurements 1 week before each experiment. SBP was measured basally, on the 2nd week, and on the 4th week. Before the measurements, rats were placed in a holder preheated to 35°C. An average value from three SBP readings (that differed by no more than 2 mmHg) was determined for each animal after they had become acclimatized to the experimental environment.

### 2.3. Blood Measurements

At the end of the 5-week period of treatment, all animals were sacrificed between 9:00 and 10:00 am by decapitation and trunk blood samples were collected to measure plasma glucose, creatinine, and uric acid. All these determinations were assayed with commercial kits implemented in an automated clinical analyzer. Testosterone was measured by *electrochemiluminescence immunoassay* (ECLIA) (Roche Diagnostics Ltd. Switzerland).

### 2.4. Renal Outcomes

#### 2.4.1. Renal Histology and Quantification of Morphology

Fixed renal tissue was embedded in paraffin and processed accordingly. Classical staining techniques were used: hematoxylin and eosin, periodic acid-Schiff (PAS), and Masson's trichrome. Twelve noncrossed fields of cortex (640 × 477 mm, 10x; 20x, and 40x) per biopsy were analyzed by light microscopy (Eclipse E 200, Nikon, Microlat) and captured with a digital camera (DS-Fi1 U2, Nikon). Evaluation and quantification was performed by an independent observer (single-blind).

#### 2.4.2. Glomerulosclerosis

Masson's trichrome-stained renal cortical sections were divided into four quadrants. Segmental and global sclerosed glomeruli were reported as a percentage of the total number of glomeruli counted in one quadrant.

#### 2.4.3. Tubulointerstitial (ti) Fibrosis

Sections were stained with Masson's trichrome. Positive blue color areas (excluding glomeruli and vessels) were analyzed in Image Pro Plus (Media Cybernetics).

#### 2.4.4. Arteriole

For each arteriole, the outline of the vessel and its internal lumen (excluding the endothelium) were generated using computer analysis (Image Pro Plus 7.0; Media Cybernetics) to calculate the total arteriolar medial area (outline/inline) in 30 arterioles per biopsy. The media/lumen (M/L) ratio was calculated by the outline/inline relationship.

### 2.5. Statistical Analysis

Values are expressed as means ± SEM. Significant differences between treatment groups were determined by two-way ANOVA. When *p* < 0.05, ANOVA posttest comparisons were made using a Bonferroni multiple-comparison test. The relationship between variables was assessed by correlation analysis. Statistical analysis was performed with Prism version 5.04 (Graph Pad Software, San Diego, CA). Also, data were analyzed by general lineal model, which in addition introduces the interaction between the factors in the model and transforms the heterogeneity of variances when, even with transformations, normality and homogeneity of variances are achieved. When a variable was observed over time, such as SBP, a random factor was introduced (hence the mixed denomination: the presence of fixed factors: treatments and the gonadal condition, and a random factor (time)). In order to analyze the differences of each random variable between the treatments and the gonadal state, the multiple comparisons method of Di Rienzo, Guzman, and Casanoves (DGC) was used, using the multivariate cluster analysis technique [19].

## 3. Results

### 3.1. Body Weight

In normogonadic animals, no significant difference was observed between the groups at the end of the different treatments. Similar results were found in the hypogonadic groups. But significant differences in the final weight were found in all groups either normogonadic or hypogonadic except for the group that only received fructose (Table 1).

### 3.2. Water Intake

All animals receiving fructose drank more liquid volume than control or animals receiving other treatments, NGN group: (C: 122.5 ± 17.5, F: 258 ± 65, OA: 107.5 ± 22.5, FOA: 250 ± 50 ml/day (*p* < 0.01)), HGN: (C: 125 ± 15, F: 235 ± 65, OA: 105 ± 5, FOA: 250 ± 50 ml/day (*p* < 0.01)).

### 3.3. Blood Pressure

All normogonadic animals had similar blood pressure at the beginning of the experiment, while hypogonadic animals showed higher levels than normogonadic ones (*p* < 0.01), with no differences in the basal values in the hypogonadotropic group (Table 2).

**Figure 1:**
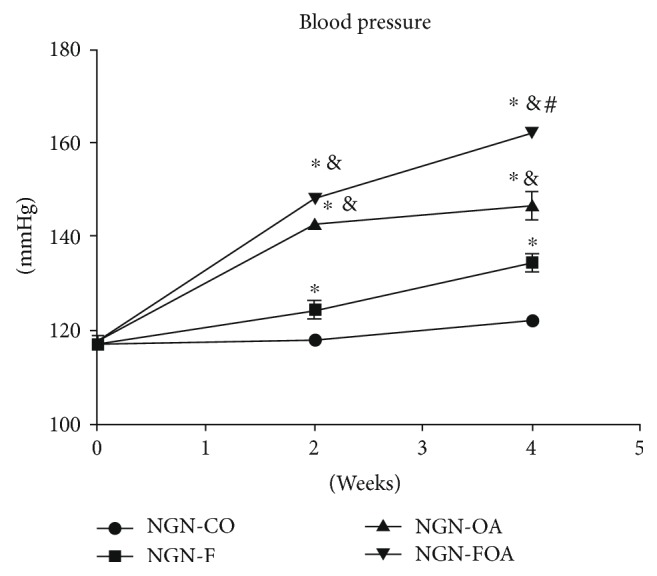
Blood pressure in NGN groups, basally and on the 2nd and 4th weeks after beginning of treatment. NGN (normogonadic); CO (control); F (fructose); OA (oxonic acid); FOA (fructose and oxonic acid). ^∗^*p* < 0.001 control group versus F, OA, and FOA groups on the 2nd and 4th weeks. ^&^*p* < 0.01 F group versus OA and FOA groups on the 2nd and 4th weeks. ^#^*p* < 0.01 OA group versus FOA groups on the 4th week.

**Figure 2:**
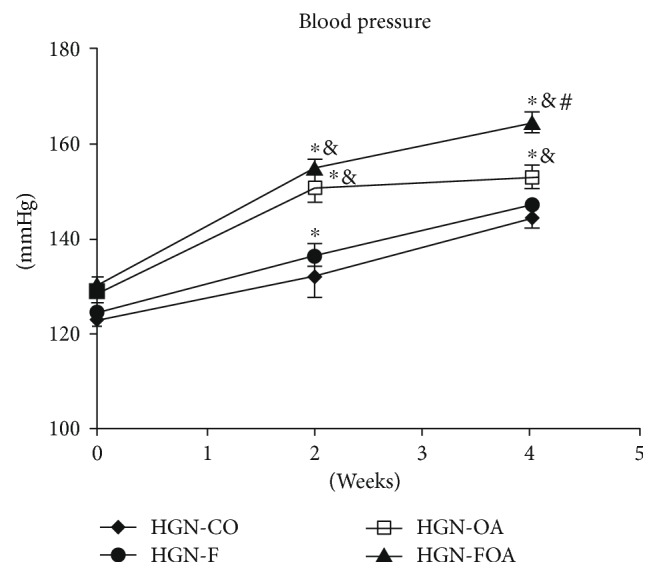
Blood pressure in HGN groups, basally and on the 2nd and 4th weeks after beginning the treatment. NGN (normogonadic); CO (control); F (fructose); OA (oxonic acid); FOA (fructose and oxonic acid). ^∗^*p* < 0.001 control group versus F, OA, and FOA groups on the 2nd week; control group versus OA and FOA groups on the 4th week. ^&^*p* < 0.01 F group versus OA and FOA groups on the 2nd and 4th weeks. ^#^*p* < 0.01 OA group versus FOA groups on the 4th week.

Two weeks of treatment passed and a significant blood pressure increase was observed in normo- (Figure 1) and hypogonadic animals (Figure 2), in the groups that received fructose, oxonic acid, or both drugs versus control groups. As it occurred with basal values, hypogonadic animals showed higher blood pressure (*p* < 0.01) than normogonadic ones (Table 2). Similar results were observed at the four-week treatment (Figures 1 and 2) but there was no significant difference between normo- and hypogonadic rats in the OA and FOA groups (Table 2).

### 3.4. Biochemical Variables

As expected, testosterone levels decrease at a very low level in all hypogonadic animals compared to all noncastrated rats (*p* < 0.03). Nevertheless, there were no differences between different drug treatments.

In both (normogonadic and hypogonadic) groups, there was no difference in plasmatic creatinine levels when comparing the treatment groups. But when comparing NGN versus HGN groups for all types of treatment, creatinine levels were higher in hypogonadal animals for all subgroups (*p* < 0.03).

Uric acid levels were significantly higher in normogonadic and hypogonadic animals treated with OA and FOA when compared with respective control groups and those groups treated only with F. Likewise, there were no significant differences when comparing NGN and HGN animals with the same treatment, even though there was a tendency found in hypogonadic animals to show lower levels compared to the normogonadic ones (Table 3).

No significant differences were observed in fasting glucose levels between normo- and hypogonadic groups with different treatments (data not shown).

### 3.5. Correlation between Testosterone and Weight, BP, and Biochemical Variables

We found a significant correlation between testosterone and weight (*r*: −0.54, *p* < 0.0001) as well as between testosterone and blood pressure (*r*: −0.44, *p* < 0.001).

### 3.6. Renal Histology

#### 3.6.1. Glomerulosclerosis

**Figure 3:**
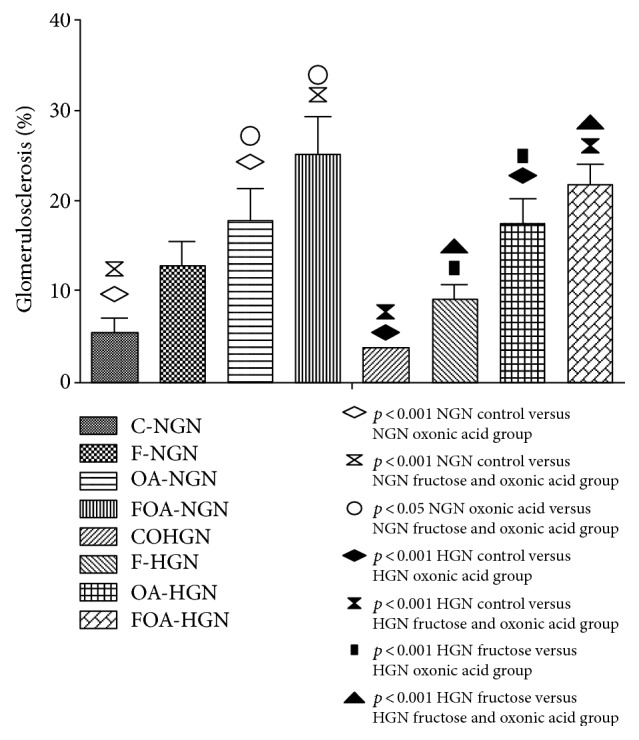
Percentage of renal glomerulosclerosis. NGN (normogonadic); HGN (hypogonadic); CO (control); F (fructose); OA (oxonic acid); FOA (fructose and oxonic acid).

**Figure 4:**
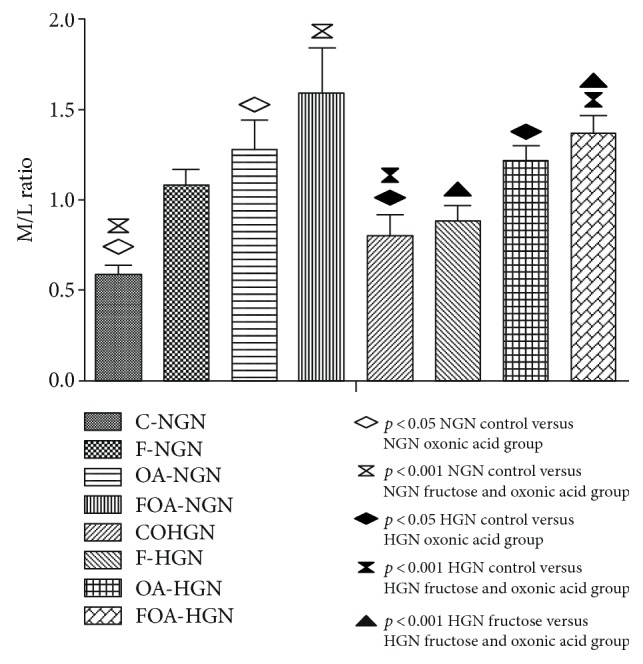
Media/lumen (M/L) ratio of renal arteriole. NGN (normogonadic); HGN (hypogonadic); C (control); F (fructose); OA (oxonic acid); FOA (fructose and oxonic acid).

In the normogonadic group, an increase in the percentage of sclerosed glomeruli was observed when comparing the experimental group to the control group. The group which only received fructose doubled the percentage of sclerosed glomeruli, but this difference was not statistically significant. The OA group significantly increased (*p* < 0.05) the percentage of glomerulosclerosis. Finally, the simultaneous exposure to fructose and OA caused a greater increase in this type of lesions (*p* < 0.001). Also, a significant increase of glomerulosclerosis was found in animals that receive simultaneous treatment (FOA) when compared to the group only receiving fructose (*p* < 0.05). In the hypogonadic group, the animals which only received fructose had the same behavior as the normogonadic with same treatment, so it was not statistically significant. Hypogonadic animals showed a significantly different glomerulosclerosis between OA and control group, which was greater than the one observed in the same normogonadic group (*p* < 0.001). The hypogonadic animals which received fructose and OA showed the same significant increase as normogonadic ones (*p* < 0.001). Also, an increase in the sclerosed glomeruli in groups OA and FOA was observed compared to animals receiving fructose (*p* < 0.05 and *p* < 0.01, resp.). Comparing all normogonadic experimental groups versus hypogonadic ones, we observed a trend to a lower glomerulosclerosis in the latter group, although this was not statistically significant (Figures 3 and 4).

#### 3.6.2. Tubulointerstitial Fibrosis

No significant difference was found between the different experimental groups or different gonadal conditions.

#### 3.6.3. Arteriole Media/Lumen (M/L) Ratio

**Figure 5:**
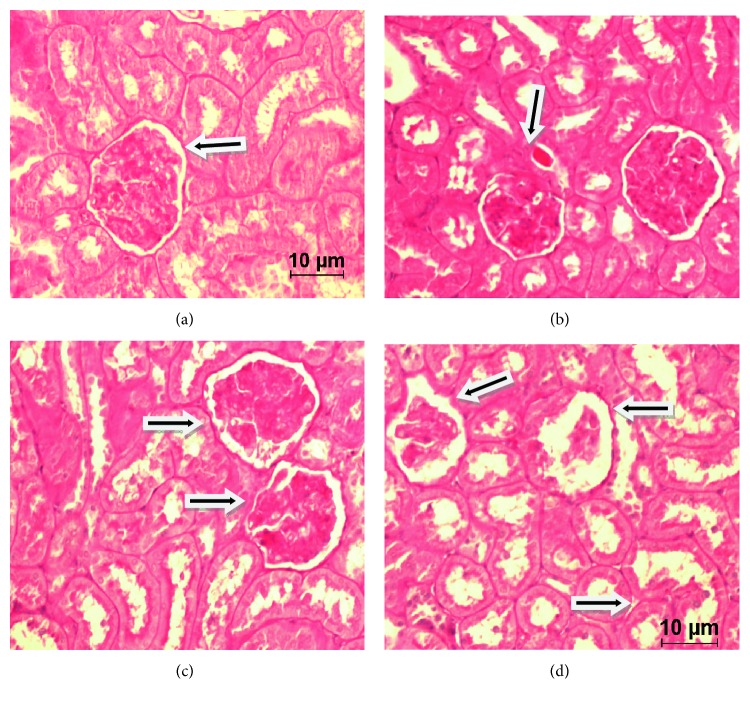
(a, b, c, d) (PAS 40x magnification. Scale bar = 10 *μ*m.) (a) Control group: normal glomeruli (arrow). (b) Fructose group: glomeruli slightly decreased in size (arrow). (c) Oxonic acid group: glomerulosclerosis (arrows). (d) Fructose and oxonic acid group: total glomerulosclerosis (arrows). These alterations were observed in both groups (normogonadic and hypogonadic rats).

In normogonadic animals, we observed a greater arteriolar M/L ratio in the groups treated with OA (*p* < 0.05) and with FOA (*p* < 0.001) when compared to the control group (C). In fructose groups, we found a strong trend to the increase of this ratio, but not reaching a statistically significant difference. In hypogonadic groups, we proved the same changes as in the previously mentioned groups (C versus OA *p* < 0.05 and C versus FOA *p* < 0.001). In addition, a statistically significant increase was observed in the fructose plus OA group versus the group only receiving fructose (*p* < 0.01). No significant difference was found between normo- and hypogonadic groups receiving similar treatment (Figure 5). No signs of acute and chronic urate nephropathy were observed in animals receiving OA and FOA.

## 4. Discussion

Fructose is a simple sugar that is present in fruits and honey and is responsible for their sweet taste. Excessive fructose intake (>50 g/d) may be one of the underlying etiologies of metabolic syndrome, type 2 diabetes, and kidney damage. One of the more striking aspects of fructose is its ability to stimulate uric acid production. As ATP is consumed, AMP accumulates and stimulates AMP deaminase, resulting in uric acid production. Researchers have reported a dose-dependent relationship between fructose ingestion and serum uric acid levels in both men and women, although in another study this relationship could not be confirmed in women [20].

It is known that chronic hyperuricemia and high-fructose intake induced features of the metabolic syndrome, including hypertension, hyperuricemia, hyperglycemia, and systemic and hepatic triglyceride accumulation. In addition, hyperuricemia alone also induced glomerular hypertension, and high fructose alone induced insulin resistance. Little is known about the action synergy of hyperuricemia and fructose-rich diets on renal function [20].

Rodents have an active uricase, and these findings explain why high concentrations of fructose are required to induce greater metabolic changes and renal disease in rats, whereas humans, who lack uricase, appear to be much more sensitive to the effects of fructose. For this reason, we induce hyperuricemia with an inhibitor of uricase, oxonic acid.

That is how during our work we found that normogonadic and hypogonadic groups treated with OA and FOA showed a higher renal glomerulosclerosis compared to control animals, and regarding the hypogonadic group a tendency to a minor lesion was observed. The animals treated with fructose during 5 weeks showed a higher glomerular lesion compared to control animals, but it was not statistically significant. An inverse relationship was described between plasmatic testosterone levels and renal lesion [21]. The lack of statistically significant differences in our results between both gonadal conditions could be explained by the short period of time passed between animal castration and sacrifice and the type of treatment carried out.

The fructose as well as uric acid share similar renal action mechanisms. The fructose enters directly the tubulus by the GLUT-5 and indirectly through the conversion of glucose into fructose through polyols, which as an end product might result in uric acid [22, 23] due to high concentration of xanthine oxidase. Different studies [24, 25] have shown that fructose accelerates the progression of tubular and glomerular lesion by cell growth, apoptosis, increase of the chemotactic protein expression of monocytes type 1 (MCP-1), endothelial dysfunction due to an increment of intracellular adhesion molecule 1 (ICAM-1), and a nitric oxide decrease. Similar effects were observed in hyperuricemia.

Uric acid can activate vascular smooth muscle cells, and this involves uptake via an organic anion transport system, activation of specific MAP kinases, stimulation of COX-2, PDGF A and C chain, and various inflammatory mediators, including C-reactive protein and monocyte chemoattractant protein-1 (MCP-1) [26]. Soluble uric acid also blocks NO release [27]; the combination of a proliferative effect on vascular smooth muscle cells and an inhibitory effect on endothelial cells likely explains why uric acid is particularly effective at causing small vessel arteriolar disease. Chronic hyperuricemia would lead to development of preglomerular vascular disease. This can be associated with activation of the renin angiotensin system and is also likely linked with endothelial dysfunction [26].

Although we have not found alterations representing final stages of tubular lesion like interstitial fibrosis, as found by different researchers [28], this could be due to the short treatment time carried out. We want to make it clear that it was not the objective of this work to evaluate tubular injuries of earlier onset.

Different hypotheses have been argued by various authors to explain the relationship between testosterone and renal damage: (a) an increase in the angiotensinogen by testosterone and stimulation of the AT-1 receptors expression due to dihydrotestosterone [29]; (b) an increase in the endothelin-1 level, strong vasoconstrictor which increases the reabsorption of renal sodium and promotes the oxidative stress [30]; (c) antioxidant enzyme inhibition by testosterone and amplification of ROS generation in response to the renal injury [31]; (d) induction of podocytes [32] and tubular cells apoptosis [33].

On the other hand, in all treatments we found an increase in uric acid levels in normogonadic animals compared to the hypogonadic ones. This could be the result of testosterone stimulant action on the expression of the urate transporter-1 (URAT-1) responsible for the reabsorption of urates at tubular level. Also the monocarboxylate transporter expression coupled with sodium types 1 and 2 which facilitates the presence of essential lactate for the urate/lactate transport by URAT-1 [34].

On the other hand, we observed an inversely significant correlation between testosterone and body weight. Studies in humans have shown that hypogonadic men have an increase in body weight and in waist circumference, and the hormone replacement improves these anthropometric parameters [35–37]. Although the mechanism has not been completely clarified, it was stated that adipocytes express androgen receptor [38] and testosterone inhibit lipoprotein lipase (LPL) activity, responsible for the uptake of triglycerides by the adipocytes, and so producing an inhibition of the triglyceride uptake and a decrease of visceral adipose tissue [39]. On the contrary, the lack of testosterone produces a higher triglyceride uptake with the subsequent increase of visceral fat. This increment favors a rise in the aromatase, increasing the conversion to estrogen. On the other hand, it favors the resistance to insulin, which results in a SHBG decrease, thus increasing the testosterone metabolism [40].

Regarding blood pressure, we observed that hypogonadic animals showed higher levels than normogonadic ones. Different studies have shown that this situation could be reverse when androgen was substituted [41, 42]. The blood pressure increase described could be partly due to the weight increase observed in these animals, as well as the resistance to insulin and cytokine increase which favors vasoconstriction. We confirmed this data showing the inversely significant correlation between testosterone and blood pressure. Also, the increase in blood pressure could be explained, as mentioned earlier, by an increase in angiotensin and endothelin, among others.

As expected, blood pressure was higher in animals treated with fructose, OA, and FOA compared to control animals in both gonadal conditions. We also found similar results regarding the arteriolar M/L ratio. As stated previously, fructose and uric acid cause renal lesion favoring formation of free radicals and glycation end products leading to vasoconstriction and endothelial lesion.

The strength of our study is to have added the gonadal state (influence of testosterone levels) to the condition of hyperuricemia and diet rich in fructose. Through this study, it is possible to emulate how the change from normogonadic to hypogonadal condition, in addition to harmful habits, can impact on the human kidney.

Our limitation lies in the socioeconomic status of a developing country, which makes it impossible to carry out other complementary techniques.

We are convinced that the results obtained in the present study will encourage the development of additional research related to the effects of the gonadal state and other conditions (hyperuricemia and diet rich in fructose) on the kidney.

## 5. Conclusion

Taking into account our objective, in short, hyperuricemia conditions as well as a high-fructose diet favor a rise in blood pressure together with vascular changes shown by the arteriolar media/lumen ratio, as well as an increase in the renal glomerular damage; these effects are more relevant in animals with both conditions simultaneously (FOA). Also, it was shown that the said changes are related to testosterone levels being more evident in normogonadic animals. It is necessary to carry out more studies to understand the mechanisms involved in such changes.

## Figures and Tables

**Table 1:** Final body weight between normogonadic and hypogonadic animals.

Final body weight (gr)	NGN	HGN	*p*
Control	373.0 ± 11.49	447.0 ± 22.78	0.01
Fructose	397.1 ± 15.47	419.5 ± 12.69	NS
Oxonic acid	358.7 ± 9.640	423.8 ± 16.15	0.004
FOA	354.7 ± 23.71	433.0 ± 18.92	0.028

Data are expressed as mean ± SEM.

NGN: normogonadic, HNG: hypogonadic, FOA: fructose and oxonic acid.

**Table 2:** Blood pressure. Comparative effect of the gonadal state at different stages of treatments.

Blood pressure (mmHg)	NGN	HGN	*p*
*Basal*			
Control	117.1 ± 1.010	122.9 ± 1.487	0.008
Fructose	117.1 ± 1.010	124.3 ± 2.296	0.015
OA	117.9 ± 1.010	132.9 ± 2.857	<0.0001
FOA	117.9 ± 1.010	142.9 ± 2.857	<0.0001
*2nd week*			
Control	117.9 ± 1.010	132.1 ± 4.738	0.012
Fructose	124.3 ± 2.020	136.4 ± 2.369	0.002
OA	142.9 ± 1.010	150.7 ± 3.168	0.036
FOA	147.9 ± 1.010	155.0 ± 1.543	0.002
*4th week*			
Control	122.1 ± 1.010	144.3 ± 2.020	<0.0001
Fructose	124.3 ± 2.020	147.1 ± 1.010	<0.0001
OA	146.4 ± 3.030	152.9 ± 2.405	NS
FOA	162.1 ± 1.010	164.3 ± 2.020	NS

Data are expressed as mean ± SEM.

NGN: normogonadic, HNG: hypogonadic, FOA: fructose and oxonic acid.

**Table 3:** Biochemical variables.

	NGN	HGN	*p*
*Plasmatic * *testosterone (ng/dl)*			
Control	2.48 ± 0.85	0.03 ± 0.007	0.039
Fructose	4.97 ± 1.37	0.035 ± 0.007	0.001
OA	4.93 ± 1.18	0.038 ± 0.007	0.001
FOA	3.44 ± 0.85	0.050 ± 0.013	0.003

*Plasmatic * *creatinine (mg/dl)*			
Control	0.444 ± 0.037	0.544 ± 0.017	0.002
Fructose	0.412 ± 0.048	0.541 ± 0.048	0.064
OA	0.424 ± 0.036	0.504 ± 0.016	0.007
FOA	0.458 ± 0.025	0.495 ± 0.012	0.026

*Plasmatic uric * *acid (mg/dl)*			
Control	0.972 ± 0.04^∗^^&^	0.962 ± 0.067^∗∗^	NS
Fructose	1.081 ± 0.061^#^	0.935 ± 0.073^&&^	NS
OA	1.275 ± 0.139^∗^	0.95 ± 0.057^##^	NS
FOA	1.491 ± 0.1^&#^	1.29 ± 0.061^∗∗^^&&##^	NS

Data are expressed as mean ± SEM. NGN: normogonadic, HNG: hypogonadic, OA: oxonic acid, FOA: fructose and oxonic acid, and NS: nonsignificant. ^∗^*p* < 0.05 NGN control versus NGN oxonic acid group. ^&^*p* < 0.01 NGN control versus NGN fructose and oxonic acid group. ^#^*p* < 0.01 NGN fructose versus NGN fructose and oxonic acid group. ^∗∗^*p* < 0.01 HGN control versus HGN fructose and oxonic acid group. ^&&^*p* < 0.01 HGN fructose versus HGN fructose and oxonic acid group. ^##^*p* < 0.01 HGN oxonic acid versus HGN fructose and oxonic acid group.
